# Supplementary material for: The Covid-19 pandemic and the expansion of the mortality gap between the United States and its European peers
Source: PLoS One. 2023 Mar 29;18(3):e0283153. doi: 10.1371/journal.pone.0283153 (PMC10057752; doi:10.1371/journal.pone.0283153)
Supplement: S1 File — (DOCX) [file pone.0283153.s001.docx]

**Supporting Information: Comparability with earlier excess-death estimates**

This study uses data on death counts and death rates for every week up to the end of 2021 in France, Germany, Italy, Spain, the United Kingdom, and the United States, by sex and five large age groups (0-14, 15-64, 65-74, 75-84 and 85+). Excess deaths are estimated by applying population-weighted averages of the sex- and age-specific rates of the European countries to US population by sex and age groups. To compare with an earlier estimate for 2017 that benefited from disaggregated data by single year of age^1^ (still unavailable for 2020 and 2021 at this writing), excess deaths were first re-estimated for 2017. To be more comparable with this earlier estimate for 2017 which derived the number of counterfactual deaths from the arithmetic (unweighted) average of the year, sex, and age-group specific death rates in each of the five European countries, estimates were derived using both the arithmetic and the population-weighted average rates.

For 2017, the only year for which the comparison is possible with the data currently available, estimates based on the data retrieved in January 2022 were quite close to the earlier estimate based on single-year-of-age data (400,732 excess deaths): 389,305 excess deaths (2.9% fewer) with the population-weighted average and 412,232 excess deaths (2.9% more) with the arithmetic average of the five European rates. Results from the 2020 Census, however, led to a revision of intercensal population estimates in April 2022. Numbers of older adults were revised downward in particular, leading to an increase in the death rates at older ages. With these updated US death rates, excess-death estimates increased (to 442,267 and 465,069 deaths) and are markedly larger than the earlier estimate based on single-year-of-age data.

**References**

1. Preston, S. H. & Vierboom, Y. C. (2021). Excess mortality in the United States in the 21^st^ century. *Proc Natl Acad Sci U S A*., 118, Article e2024850118. https://www.pnas.org/doi/10.1073/pnas.2024850118
