# Supplementary material for: The Covid-19 pandemic and the expansion of the mortality gap between the United States and its European peers
Source: PLoS One. 2023 Mar 29;18(3):e0283153. doi: 10.1371/journal.pone.0283153 (PMC10057752; doi:10.1371/journal.pone.0283153)
Supplement: Attachment — Submitted filename: Response to Reviewers.docx [file pone.0283153.s002.docx]

Response to Reviewers’ comments (descriptive sentences that did not call for revisions were edited out).

Reviewer #1: 
*The analysis is mostly sound however I have a few queries about the choice of some of the measurements used,
The title of the paper and a major finding is that the annual number of excess deaths in the US versus the 5 European countries doubled between 2017 and 2021. This finding is based on the total number of excess deaths in the first row of Table 1 – which increased from 442,267 in 2017 to 892,491 in 2021. However, that measure includes the contribution of population change to the excess deaths of 42,317 (shown lower down in Table 1). Population change should be standardised for in analysis of trends in the number of excess deaths because it does not measure changes in population health but rather demographic trends (i.e. relative increase of the population). It would be more appropriate to compare the 442,267 figure in 2017 to 850,174 in 2021 that was standardised with the 2017 population – that is, the number of excess deaths did not double, but still increased substantially. The title and main findings of the paper should be adjusted to use the population-standardised excess mortality figures. Also, the “share of change in excess deaths” and other calculations in the manuscript should also be calculated based on population-standardised excess mortality.*

The title has been revised to refer to “the expansion of the mortality gap (no reference to a doubling).

The text now refers to population-standardized numbers of excess deaths (holding population size constant at its 2021 value rather than its 2017 value so that the 2021 numbers would be the actual number). These numbers are shown in the second row of Table 1 and the decomposition by cause (share of change in excess deaths) now refers to those.

*The calculation of the excess deaths % is confusing. Conventionally, the excess mortality % is = (number deaths pop A – number deaths pop B) / number deaths pop B. However this paper divides excess deaths by the number of deaths in population A. It would be more insightful – and would also better demonstrate the large differences in mortality – if the former more conventional calculation were used. For example, the excess deaths in 2021 of 25.8% would instead be 25.8%/(100%-25.8%) = 34.8%, which is a more intuitive figure that shows that death rates in the US in 2021 were 35% higher than for 5 European countries. For ages 15-64 in Table 2, it would show that death rates in the US are almost double that in the 5 European countries.*

Instead of share of total deaths that are excess deaths, the paper (in the text and Tables) now refers to the proportional increase in the total number of deaths caused by excess mortality, as suggested, except in the last paragraph in order to make the statistics comparable those in the cited studies..

*Figure 1 would be more intuitive for the reader if simply the results were shown as a % as the quarterly results shown, rather than rolling annual figures presented.*

Both 3-month and 12-month figures have been calculated, but seasonality makes the trend in 3-month figures more difficult to read and less meaningful that the trend in 12-month figures.

*The discussion of findings is quite limited and could point in the direction of future research to better understand why there has been a dramatic increase mortality in the US versus Western Europe – for example, measurement of the contribution of specific causes of death to the increase in excess mortality (in addition to Covid). It would also be good to describe some of the comorbidities of Covid mentioned in the 2nd paragraph of the Discussion, as well as mention differences in testing of Covid that might affect Covid death rates.*

This is largely a technical note bringing attention to the expansion of this mortality gap, and to the specific role of Covid-19 mortality, hoping to generate interest in future work on understanding its causes that is somewhat beyond the author’s expertise. References are provided to past and recent work in this area (including the addition of references 1 to 6, see below).

*Some other comments:
- In the Abstract, it states “Applying average mortality rates…” – should this be “population-weighted average mortality rates…”?*

Yes, this has been corrected.

*- There should be a section title for the Introduction*

Section title has been added.

*- The first sentence “… is well documented” should have reference(s).*

References 1 to 6 have been added.

*- Delete “so” from “the annual number of excess deaths estimated so nearly doubled from…”*

Deleted.

*- I had trouble finding the data for “Relatively stable until mid-2021 (from 34.6% for 2020 to 37.2% for the mid-2020 to mid-2021 period), that fraction decreased to 29.2% in the 12-month period ending on October 1st, 2021, before bouncing back to 48.2% for the calendar year 2021.”*

The percentages refer to the ratio of excess deaths to total deaths among those involving Covid-19 (height of the shaded part of the bar to height of the entire bar) in Figure 2. These percentages have been added to the Figure.

*- Suggest choosing another word other that “impressive” to describe the increase in excess deaths – e.g. “substantial”*

“Impressive” has been replaced with “marked.”

Reviewer #2:

*One main concern is that the report should be more explicit in the unique contribution of this analysis over the Heuveline (2022) PNAS paper. A few sentences in the Introduction would suffice.*

This description was added at the end of the “Introduction” Section: “This study expands on these previous studies by extending the estimation of the annual number of US excess deaths relative to the same five European countries from 2017 to 2021, and of the specific contribution of differences in Covid-19 mortality from April 1, 2021 to the end of 2021.”

*The methodology should also be made more explicit.*

The methodology was made more explicit in the “Material and methods” Section, which now includes the step-by-step equations used to derived the numbers in Tables 1 and 2 and in Figure 1.

Specific comments:
*1. The gap in mortality between the U.S. and Europe has been long-standing, albeit growing. I don’t think “emergence” is the appropriate term as it implies that the gap emerged in the years leading up to the pandemic.*

Title, abstract and first sentence now refer to an “expansion” of the mortality gap.

*2. It might be useful to add a row for “Total Deaths” to Table 1 since percentage excess deaths are based on total recorded deaths.*

Total deaths are now shown in the first row of Table 1.

*3. The terms “ratio” and “share” are used in Table 1 – but percentages are shown in the Table. It may be helpful to use “Percent Excess Deaths” or “Percent Contribution…” as appropriate.*

“Percentage” is now used in Table 1.

*4. COVID-19 deaths: Were these measured by counting deaths with COVID-19 on the death certificate or calculations of excess deaths attributable to COVID-19? The method should be made clearer in this regard.*

Methodological details added in the “Material and methods” Section now indicate that those deaths are “deaths involving Covid-19” as reported by the CDC.

*5. What is the explicand for the cause of death contributions? Is it the increase in excess deaths between each year and 2017? Is it the excess deaths for a given year? It is also not clear if the contributions are calculated with respect to the age-sex standardized excess deaths (or change in excess deaths).*

Table 1 rows as now labelled as “Percentage of standardized number of excess deaths from all causes” to clarify that the contribution is to the population-standardized excess deaths for a given year (shown in a row above in Table 1).

While revising your submission, please upload your figure files to the Preflight Analysis and Conversion Engine (PACE) digital diagnostic tool, [https://pacev2.apexcovantage.com/](https://linkprotect.cudasvc.com/url?a=https%3a%2f%2fpacev2.apexcovantage.com%2f&c=E,1,x9a57ZFAcfVYBRfdaFBGHd3QS57BkWUxaBhSNMddIYNDNX99KiFuvwOK6Ifpu_mOz72aL8t1mQcoMc7HlkFUtu-8dJ0i17ne_b8lwTn_JE-5pbdXCw,,&typo=1). PACE helps ensure that figures meet PLOS requirements. To use PACE, you must first register as a user. Registration is free. Then, login and navigate to the UPLOAD tab, where you will find detailed instructions on how to use the tool. If you encounter any issues or have any questions when using PACE, please email PLOS at [figures@plos.org](mailto:figures@plos.org). Please note that Supporting Information files do not need this step.
